# The image of mind in the language of children with autism

**DOI:** 10.3389/fpsyg.2015.00841

**Published:** 2015-06-18

**Authors:** Wolfram Hinzen, Joana Rosselló, Otávio Mattos, Kristen Schroeder, Elisabet Vila

**Affiliations:** ^1^Institució Catalana de Recerca i Estudis Avançats (ICREA)Barcelona, Spain; ^2^Department of Philosophy, University of DurhamDurham, UK; ^3^Grammar and Cognition Lab, Department of Linguistics, Universitat de BarcelonaBarcelona, Spain

**Keywords:** autism, language, grammar, reference, deixis, pronouns, communication

While it is widely agreed today that autism involves a cognitive change (Baron-Cohen, 1988), the main psychological models have put the explanatory weight on changes in such non-linguistic neurocognitive variables as “theory of mind” (ToM), weak central coherence, or executive functioning. Linguistic deficits, including ones identified as “pragmatic” and taken to be universal in people with autism spectrum disorders (ASD) (Tager-Flusberg, 1996; Lord and Paul, 1997; Tager-Flusberg et al., 2001), or even the absence of functional language could then be seen as a secondary consequence of a primary defect in non-linguistic (particularly social) cognition (Mundy and Markus, 1997). A “modular” perspective, which separates language from cognition, has been widely adopted with regard to the internal organization of language itself, which is taken to comprise phonology, syntax, semantics, and pragmatics as relatively independent components. In this regard, Tager-Flusberg (1981) formulates the classical view that “phonological and syntactic development follow the same course as in normal children and in other disordered groups, though at a slowed rate, while semantic and pragmatic functioning may be specially deficient in autism.”

More recently, attention was drawn to a potential subtype of ASD, autism with language impairment (ALI), showing deficits in structural aspects of language comparable to those in Specific Language Impairment (SLI) (Tager-Flusberg and Joseph, 2003), additionally to standard impairments in social interaction and communication, and behavioral abnormalities. Specific deficits in such domains as non-word repetition (phonology) or verbal inflection (morphology), as seen in this subtype (Tager-Flusberg, 2006), however, provide little basis for understanding the distinctive ASD cognitive “style” (Happé and Frith, 2009). Indeed the *absence* of co-morbid non-verbal cognitive impairment is a defining feature of SLI.

Yet it is not clear how independent of language human-specific *forms* of social interaction and communication can be, and it could be that a fundamental alteration in language competence is an inherent *aspect* of the cognitive change in question. Children with autism might *construe* language differently, reflecting a linguistic style different from that inherent in neurotypical cognition, which could then be reflected in altered patterns of social communication. Rapin and Dunn (2003) already suggested, not only that phonology and syntax are impaired in autism, but that there is a relation between phonological and syntactic deficits, and between semantic and pragmatic ones. Kjelgaard and Tager-Flusberg (2001, p. 10) suggest that phonological deficits are only present in those children with higher-order semantic and syntax deficits.

Language could matter in a different way, if it was more than communication—and more even than a human-specific style of communication: It could also be *cognition*, i.e., a principle for having the particular *type* of thought that is communicated in language (Hinzen and Sheehan, 2013). It is surely not simply an accident that humans, apart from having the cognitive type that they do, also speak: subtracting our linguistic capacity from our cognitive toolkit does not allow the same mind to develop, though now it is now tragically speechless. Language makes us social in ways that no other species is, allowing us a form of communication in which propositional thoughts can be articulated and shared. A look at the cognitive types and communications of non-linguistic species (Hauser, 1996; Penn et al., 2008; Tomasello, 2008), and at the cultural productions of extinct hominins (Tattersall, 2008), suggests an explanatory gap between species with and without a language-like symbolic code. Language remains one of the most likely cognitive principles for explaining what transformed pre-sapiens cognitive phenotypes into their modern human variety, re-configuring the hominin mind rather than merely expressing one that pre-existed the arrival of linguistic communication (Tattersall, 2014). Hinzen and Sheehan (2013) describe the organizational principles of language *as* those of human-specific thought.

In line with this linguistic model, we also see language playing an intrinsic role in the development of normal thought and communication in infants (Vouloumanos and Waxman, 2014). Where there is no exposure to speech/sign, or language does not develop normally, thought is diminished or altered as well, as in language-less adults (Schaller, 2012), deaf children deprived of a sign language (Humphries et al., 2014), or children with autism, as we argue below. Arguably our relationship to ourselves in general, i.e., our human-specific sense of self, is mediated by language and narrative, in that we canonically refer to ourselves in the grammatical 1st Person (“I”), connecting linguistically to others who are grammatical 2nd Persons for us (“you”). What we say to them in turn carries information about a world viewed as distinct from both of us and the thoughts we share: it is grammatically the 3rd (or non-) Person (“it,” “s/he,” “that”). This threefold distinction of grammatical Person spans a “deictic space” in which all human thought and communication is anchored or embedded. This space is partially human-specific and Person distinctions are defined in relation to when speech acts take place, making a relation not only to language but to speech/sign inherent. Saying “I” as and when we speak, we locate ourselves in this space, with a past that partially defines us, a future which language helps us to creatively construct, and a personal narrative involving others. We argue in the remainder that a disturbance in this *language-mediated deictic frame* could play a key role in making sense of the autism cognitive and communicative phenotype, providing a unified linguistic and psychological model.

That language is indeed construed differently in ASD is strongly suggested by the universality of both communicative and pragmatic impairments in the autism spectrum, while the status of repetitive behaviors and interests is more contested (Mandy and Skuse, 2008). It could only be by *separating* a “pragmatic” or a “communication” impairment from a “language impairment,” that the claim of a language impairment in only a *subgroup* of individuals could be upheld. In general, language and human-specific communication can barely be separated. Our position predicts that language in ASD should not merely be delayed or slowed but *altered*, in ways that illuminate the cognitive change, and a significant decrease in general *syntactic* ability across children with ASD as compared with children with developmental delays or mental retardation has now been documented (Pierce and Bartolucci, 1977; Eigsti et al., 2007). In Eigsti et al., 2007, syntactic ability was negatively correlated both with use of jargon/echolalia and with presentism/concretism (lack of displacement). Beyond delayed onset of first words as also present in other disorders, children with ASD can show a deviant pattern of lexical growth (Lord et al., 2004).

As for problems with communication, the problem in ASD does not lie in communication as such—children with ASD can and do *communicate*—but in the normal forms of *linguistic* communication (Maljaars et al., 2011). There are basic disturbances in speech production and perception that could have resounding effects in the contents communicated in speech. Alterations in speech perception (Alcantara et al., 2004) and the preference of speech over non-speech (Klin, 1991) have been documented and neurophysiological methods have revealed a “profoundly different stimulus-processing manner in autism” partially specific to sound discrimination in speech (Kujala et al., 2013). Speech (or sign), the vehicle of linguistic communication, which preferentially captures newborn infants' attention from birth (Vouloumanos and Waxman, 2014), does not attract autistic children as much. Typically developing 6-month-old infants grasp the abstract role of speech in communication before having knowledge of words (Vouloumanos et al., 2014), which must further enhance their development, whether of language or thinking. By contrast, even siblings at high risk for ASD at 12 months do not pay the same attention to speech as typically developing children (Curtin and Vouloumanos, 2013). Deficits of prosody are also common (Peppe et al., 2007).

Furthermore, supposedly “non-verbal” aspects of cognitive development such as joint attention or pointing appear to be inherently linked to linguistic development. A tight coupling of declarative pointing and language has been demonstrated in normal children (Cartmill et al., 2014). Mundy et al. (1990), in a longitudinal study of early joint attention and language, found that the former was a significant predictor of the latter in autistic children. Joint attention seems to be crucially enhanced by a bias toward speech already present in newborn infants (Vouloumanos and Waxman, 2014), and it is an ingredient of declarative pointing, which is clearly impaired in ASD. Declarative pointing is co-morbid with a structural change in the deictic use of language more generally, manifest in misuses of such expressions as “here” vs. “there” and “this” vs. “that” (Hobson et al., 2010; Hobson and Meyer, 2005).

Regarding the contents of communication, the normal declarative use of language for the purpose of affirming and sharing thoughts is deviant in verbal children with autism (Loveland et al., 1988). This is neither merely a communicative nor a pragmatic impairment, but also concerns the distribution of linguistic forms: imperatives cannot, in virtue of their form, encode declarations, while declaratives can express commands only via implicatures. Maljaars et al. (2011) found that among three forms of communication, namely behavior regulation, social interaction, and joint attention, the proportion of the first to the third was much higher in children with autism than in typically developing children, who showed the opposite pattern. The non-verbal group with autism did not or barely communicate for declarative purposes and used the least complex forms of communication.

Part and parcel of the general problems with deixis are classical misuses of pronouns, as in the non-shifted pronoun use resulting from echolalia but also the spontaneous substitutions of non-standard grammatical Persons for where personal (1st or 2nd) forms would be expected. In a recent corpus-based study of two French-speaking autistic children, Dascalu (2014) found that non-standard (2nd and 3rd) personal forms of self-reference and the normally expected 1st personal forms can seem context-equivalent to the autistic children. One child used French “il” (“he”) as a “passe-partout” referential device fitting all objects of reference equally. Grammatical Person was not merely distorted, moreover, but there is a distinctive *Person-shift* from personal to 3rd-personal forms for self-reference, while the reverse is not the case. As these findings suggest, not use of pronouns *as such* is impaired, but grammatical Person is. This does not appear to be the consequence of a general problem in “perspective-taking” in some non-linguistic sense, since the children did take the perspectives of others, as in numerous examples documenting that they are pursuing the trains of thoughts of others, imitate others, and role-play. The Person shift in its grammatical dimension is also confirmed by numerous earlier studies showing a preference of 3rd Personal forms of self-reference (such as the child's own proper name) in both spoken (Jordan, 1989; Lee and Hobson, 1994; Mizuno et al., 2011) and signed language (Shield and Meier, 2014); and by the fact that it does not occur in isolation but as part of a larger disturbance in the referential use of language. Thus Modyanova (2009) showed, in a comparison of indefinite NPs (e.g., “an/another apple”) with definite NPs (“the/that apple”), that children with autism compared with Asperger's or Pervasive Developmental Disorder (PDD-NOS) showed no knowledge of definite determiners at all (Lord and Rutter, 1994).

In sum, the evidence synthesized here suggests a seismic shift in grammar-based semantics and the deictic space in which all thought, speech, and reference takes place. The language change we see concerns *core* linguistic functions that depend on specific forms of grammatical organization, such as Person, determiners, or sentential complexity, though to different degrees. These functions mediate cognitive ones, and they specifically concern how language normally mediates a species-specific deictic frame. It is not clear which *non-linguistic* deficit would account for this change, when it is linguistically highly specific, and ToM in particular has itself long been noted to be strongly *correlated* with language in its explicit form (DeVilliers, 2007; Paynter and Peterson, 2010). This motivates casting a novel linguistic eye on mental change, in line with recent developments in linguistic theory that see linguistic organization as a central principle for a human-specific cognitive type and self (Hinzen and Sheehan, 2013).

## Conflict of interest statement

The authors declare that the research was conducted in the absence of any commercial or financial relationships that could be construed as a potential conflict of interest.
